# ReSETting PP2A tumour suppressor activity in blast crisis and imatinib-resistant chronic myelogenous leukaemia

**DOI:** 10.1038/sj.bjc.6603317

**Published:** 2006-09-05

**Authors:** D Perrotti, P Neviani

**Affiliations:** 1 Human Cancer Genetics Program, Department of Molecular Virology, Immunology and Medical Genetics, and The Comprehensive Cancer Center, The Ohio State University, Columbus, OH 43210, USA

**Keywords:** PP2A, SET, BCR/ABL, CML, forskolin, leukaemia

## Abstract

The deregulated kinase activity of p210-BCR/ABL oncoproteins, hallmark of chronic myelogenous leukaemia (CML), induces and sustains the leukaemic phenotype, and contributes to disease progression. Imatinib mesylate, a BCR/ABL kinase inhibitor, is effective in most of chronic phase CML patients. However, a significant percentage of CML patients develop resistance to imatinib and/or still progresses to blast crisis, a disease stage that is often refractory to imatinib therapy. Furthermore, there is compelling evidence indicating that the CML leukaemia stem cell is also resistant to imatinib. Thus, there is still a need for new drugs that, if combined with imatinib, will decrease the rate of relapse, fully overcome imatinib resistance and prevent blastic transformation of CML. We recently reported that the activity of the tumour suppressor protein phosphatase 2A (PP2A) is markedly inhibited in blast crisis CML patient cells and that molecular or pharmacologic re-activation of PP2A phosphatase led to growth suppression, enhanced apoptosis, impaired clonogenic potential and decreased *in vivo* leukaemogenesis of imatinib-sensitive and -resistant (T315I included) CML-BC patient cells and/or BCR/ABL^+^ myeloid progenitor cell lines. Thus, the combination of PP2A phosphatase-activating and BCR/ABL kinase-inhibiting drugs may represent a powerful therapeutic strategy for blast crisis CML patients.

The equilibrium between kinase and phosphatase signals is essential for the correct control of normal cell survival, proliferation and differentiation (Hunter, 1995). In fact, aberrant activity of cellular kinases has been detected in many cancers, including leukaemias. Among the different oncogenic kinase-driven neoplasia in which altered protein phosphorylation represents the molecular hallmark of the disease, the p210-BCR/ABL oncoproteins of the chronic myelogenous leukaemia (CML), can be considered the best example of an oncogene with constitutive tyrosine kinase activity (Calabretta and Perrotti, 2004).

## CML, BCR/ABL AND IMATINIB

**Figure 1:**
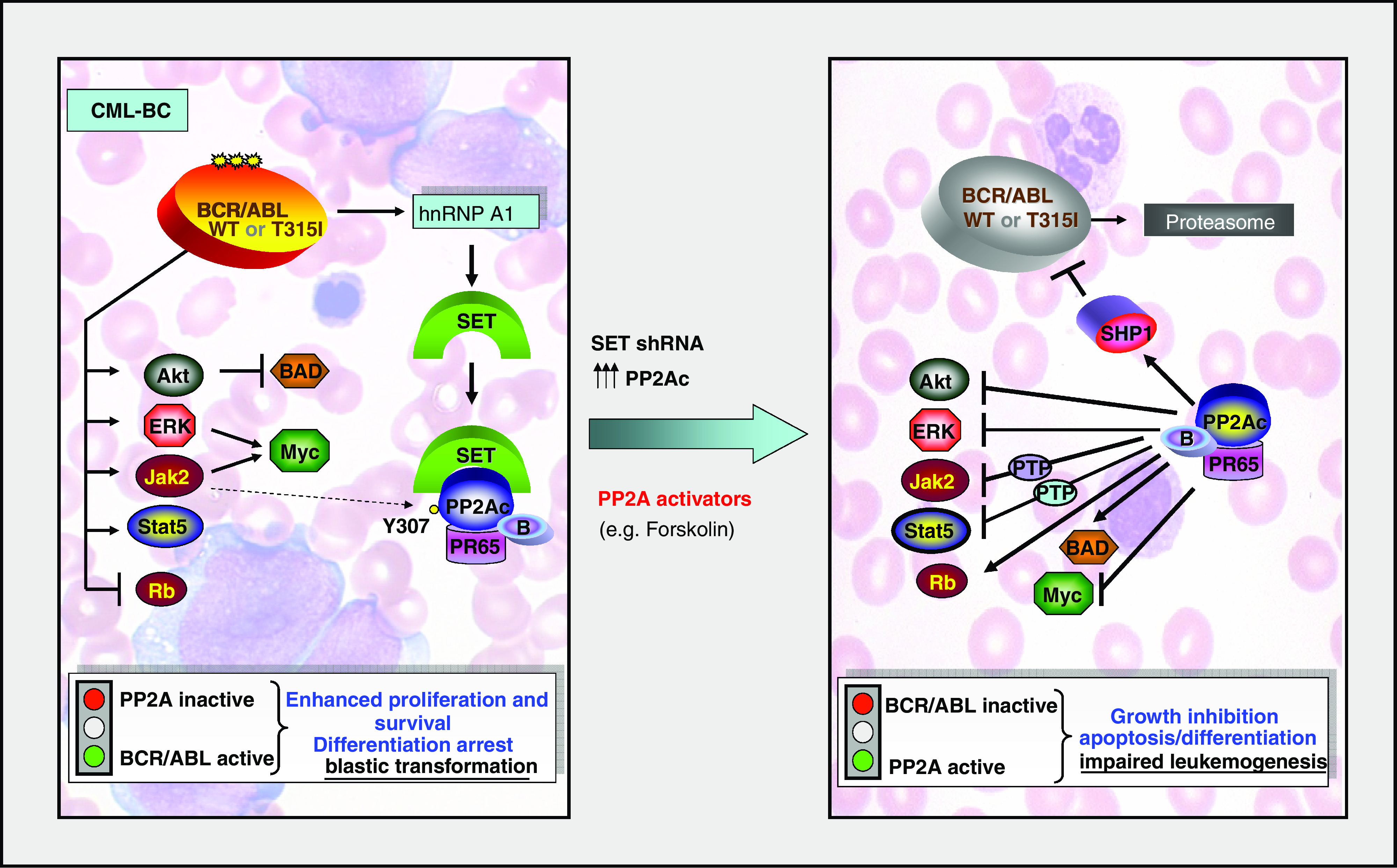
Molecular Effects of PP2A activation on BCR/ABL and its downstream effectors. (Left) BCR/ABL inhibits PP2A activity by (**A**) inducing hnRNP A1 that, in turn, enhances SET expression, and (**B**) inducing PP2Ac Y307 phosphorylation. In BCR/ABL^+^ myeloid progenitor cells, suppression of PP2A phosphatase activity is required for sustained activation of mitogenic and survival signals, in part, mediated by the indicated BCR/ABL downstream effectors. (Right) Restored PP2A activity, achieved by SET downregulation, PP2Ac overexpression or treatment with PP2A activators (e.g. forskolin or 1,9-dideoxy-forskolin), impairs *in vitro* and *in vivo* wild-type and T315I BCR/ABL leukaemogenesis by antagonising the effects of BCR/ABL on its downstream signal transducers and promoting BCR/ABL inactivation and proteasome-dependent degradation.

CML is a clonal disorder of the pluripotent haematopoietic stem cells and is clinically characterised by two distinct stages, a protracted myeloproliferative disorder termed chronic phase (CML-CP) that progresses to a rapidly fatal blast crisis (CML-BC), which can have either myeloid or lymphoid features (Calabretta and Perrotti, 2004). Hallmark of CML is the presence of the Philadelphia chromosome (Ph^1^), product of the reciprocal translocation between chromosome 9 and chromosome 22, t(9;22)(q34;q11). The translocation results in the fusion of the BCR to the c-ABL gene. In CML, the BCR/ABL fusion gene encodes for a 210 kDa protein designated as p210^BCR/ABL^. It has been well established that the sole expression of p210-BCR/ABL is sufficient for the induction of chronic phase CML, and growing evidence gives to BCR/ABL a central role also in CML progression into blast crisis (reviewed by Calabretta and Perrotti 2004). In fact, along with other frequent genetic and molecular abnormalities (e.g. double Ph^1^ chromosome, p53 inactivation) (Johansson *et al*, 2002), increased expression and activity of the BCR/ABL oncoprotein is frequently observed during CML disease progression and in blast crisis CML (Elmaagacli *et al*, 2000; Jamieson *et al*, 2004; Barnes *et al*, 2005b), and sustained BCR/ABL expression in myeloid progenitor cell lines induces phenotypic changes (i.e. differentiation arrest) characteristic of CML-BC (Perrotti *et al*, 2002). The ability of BCR/ABL to induce and sustain the leukaemic phenotype depends on its unrestrained tyrosine kinase activity (reviewed by Calabretta and Perrotti, 2004), which is essential for the recruitment and activation of multiple pathways that transduce oncogenic signals leading to growth factor-independent proliferation, increased survival and altered differentiation (Perrotti *et al*, 2002) of myeloid precursors. In fact, the pleiotropic effect of BCR/ABL transformation is mostly dependent on post-translational modifications (e.g. phosphorylation) of signalling molecules, like those involved in the RAS/MAPK, PI3K/Akt and STATs pathways, which control cell growth, survival and differentiation of haematopoietic cells by modulating the expression and/or activity of downstream effectors (Melo and Deininger, 2004; Perrotti *et al*, 2005) (Figure 1). In blast crisis, increased expression of the BCR/ABL oncoprotein accounts for the block of differentiation, inactivation of factors with tumour suppressor activity and decreased genomic stability of the Ph^1^ blasts (Perrotti *et al*, 2002; Skorski, 2002; Trotta *et al*, 2003; Calabretta and Perrotti, 2004; Neviani *et al*, 2005). Thus, dependence on BCR/ABL expression is not only a characteristic of CML-CP but also of CML-BC; however, BCR/ABL-independent mechanisms also seem to contribute to disease progression and imatinib resistance in some CML cases (Donato *et al*, 2003; Dai *et al*, 2004).

Being the deregulated BCR/ABL kinase activity the cause of CML, targeting its catalytic domain was the most rationale approach for the rational development of small molecules that inhibit ABL kinase activity. Imatinib mesylate (Gleevec, Novartis Basel, Switzerland; STI571), a Kit, Abl and PDGFR inhibitor, induces apoptosis of the Ph^1^ CML progenitors by suppressing the ability of BCR/ABL to phosphorylate substrates through competitive inhibition at the BCR/ABL ATP binding site (Druker *et al*, 1996). The development of the BCR/ABL tyrosine kinase inhibitor imatinib mesylate (Gleevec; formerly STI571) as the treatment of choice for chronic phase CML and its remarkable therapeutic effects suggest that blast crisis transition will be postponed for several years in the majority of CML patients (Deininger *et al*, 2005a; Roy *et al*, 2006). However, the persistence of BCR/ABL transcripts in a cohort of patients with complete cytogenetic response (Hughes *et al*, 2003) and the resistance of the primitive CML stem cell to imatinib treatment (Copland *et al*, 2006) raises the possibility that treatment with imatinib alone might delay but not prevent disease progression. Furthermore, most of the CML patients in the accelerated and blastic phases of the disease are either refractory or develop resistance to imatinib monotherapy (Deininger *et al*, 2005a). In these CML-BC patients, imatinib resistance often depends on reactivation of BCR/ABL tyrosine kinase activity via mechanisms involving BCR/ABL overexpression, gene amplification or mutations that suppress imatinib-mediated kinase inhibition (i.e. E255V and G250E) or disrupt imatinib binding (i.e. T315I) (Shah and Sawyers, 2003). Thus, development of imatinib resistance appears to predispose to blastic transformation. Although new phase 1 clinical trials with the dual Src/Abl inhibitor dasatinib (BMS-354825) and the selective Abl inhibitor AMN107 show encouraging results (O'Hare *et al*, 2005), as they suppress the activity of most BCR/ABL mutants (except T315I) (O'Hare *et al*, 2005), *in vitro* evidence suggests that resistance to these new compounds may develop through mechanisms involving the selection and expansion of BCR/ABL^+^ cell clones carrying the T315I BCR/ABL mutant (Deininger *et al*, 2005b). Additionally, dasatinib, like imatinib, is not effective in the treatment of CML-BC patients (Talpaz *et al*, 2006), and in killing the most primitive quiescent CML cells (Copland *et al*, 2006) and, therefore, it may also be ineffective in preventing disease progression.

The mechanisms responsible for transition of CML chronic phase into blast crisis remain poorly understood, although a reasonable assumption is that the unrestrained activity of BCR/ABL in haematopoietic stem/progenitor cells is the primary determinant of disease progression. However, a plausible model of disease progression predicts that increased BCR/ABL expression promotes the secondary molecular and chromosomal changes essential for the expansion of cell clones with increasingly malignant characteristics, and remains crucial for the malignant phenotype even in advanced stages of the disease (Calabretta and Perrotti, 2004). According to this model, CML blast crisis would be expected to occur only in patients with an imatinib-resistant disease or in those developing resistance during treatment. Indeed, a recent study from the GIMEMA Working Party on CML reported that the early detection of BCR/ABL mutations in CML chronic phase patients is associated with a greater likelihood of disease progression (Soverini *et al*, 2005). Interestingly, a direct correlation also seems to exist between levels of BCR/ABL activity and development of imatinib resistance (Schultheis *et al*, 2005; Barnes *et al*, 2005a). Thus, there is still a need for new drugs that, if used in combination with the available kinase inhibitors, will decrease the rate of relapse, prevent blastic transformation of CML and, perhaps, overcome imatinib resistance. Therefore, a better understanding of the biology of CML-BC is necessary to identify molecular networks that, if appropriately modulated, will simultaneously affect the function of BCR/ABL and that of multiple signals aberrantly activated in CML-BC.

## PROTEIN PHOSPHATASE 2A (PP2A), A PHOSPHATASE WITH TUMOUR SUPPRESSOR ACTIVITY

Protein phosphatases participate in the normal cell homeostasis by dephosphorylating substrates, therefore providing a negative feedback to signals triggered by kinases. Altered phosphatase and/or kinase activity is often associated to malignant transformation (Hunter, 1995; Blume-Jensen and Hunter, 2001). Hence, some protein phosphatases have been classified as tumour suppressors by virtue of their central role in the regulation of cell growth, survival and/or differentiation (Parsons, 1998; Janssens *et al*, 2005; Ostman *et al*, 2006).

PP2A, one of the major cellular serine-threonine phosphatase activity, is a heterotrimeric complex typically consisting of a catalytic subunit C, a structural subunit PR65/A, which exists in the isoforms *α* and *β*, and an array of different subunit B (divided into four families B, B′, B′′ and B′′′) that confers substrate specificity and target the enzyme to different intracellular location (Janssens and Goris, 2001; Schonthal, 2001). Moreover, the association of the catalytic and structural subunit with some B isoforms determines the tissue- and developmental stage-specific expression patterns to different PP2A complexes (Martens *et al*, 2004). PP2A is involved in almost all of the cellular processes, including signal-transduction pathways transducing mitogenic and survival signals (e.g. PP2A negatively regulates the RAS/MAPK and the PI3K/Akt pathways), transcriptional and translational regulation, DNA replication and control of cell cycle (Janssens and Goris, 2001; Sontag, 2001).

The ability of PP2A to antagonise cancer development has first been suggested in the late 1980s when it was described as target of the tumour promoter okadaic acid; however, only recently, PP2A received the designation of ‘tumour suppressor’ (Van Hoof and Goris, 2004; Janssens *et al*, 2005). In fact, suppression of PP2A activity appears to be a common event in different human neoplasia. For example, genetic alterations of the genes encoding the *β*-isoform of the structural subunit PP2A_A_ and some of the regulatory PP2A_B_ subunits have been found in several types of human cancer (Van Hoof and Goris, 2004; Janssens *et al*, 2005, and references therein). Moreover, oncogenic viral proteins, such as the SV40 virus small-t antigen, inhibit PP2A activity by interacting with the subunit A of PP2A complexes and displacing various B subunits (Sontag, 2001). Conversely, overexpression of PP2Ac reduces Ha-RAS-induced cellular transformation (Baharians and Schonthal, 1999). As result, loss of PP2A activity leads to constitutive activation and increased stability of protein kinases transducing mitogenic and survival signals (e.g. MAPK and AKT) and protoncogenes (e.g. Myc) (Sontag, 2001; Yuan *et al*, 2002; Yeh *et al*, 2004). Furthermore, the PP2A subunit A *per se* also has been described to be genetically or functionally altered in different types of cancer. Indeed, mutations of PR65/A*β* have been reported in primary lung tumours and in colorectal carcinomas, in which a gene deletion generates a truncated A subunit that is unable to interact with the PP2Ac catalytic subunit (Wang *et al*, 1998). Similarly, loss or reduced expression of PR65 has been detected in human gliomas and breast cancer cell lines (Colella *et al*, 2001; Suzuki and Takahashi, 2003). Accordingly, in immortalised but not tumorigenic cells, expression of PR65/A*α* mutants defective in binding to other PP2A subunits partially suppressed endogenous PP2A-A*α* expression, activated the AKT pathway and promoted tumour formation in immunodeficient mice (Chen *et al*, 2005b). Thus, mutations of the PR65/A subunits might contribute to cancer development by altering the PP2A multimeric composition and, therefore, its phosphatase activity. Alterations in the quaternary structure of PP2A also appears to have a crucial function in cells transformation and tumour development, as different B subunits have been found either aberrantly expressed in human cancers or displaced by the interaction of PR65/A with the SV40 small-t antigen, and by mutations of the PP2A A subunits (Janssens *et al*, 2005). For example, the B56*γ* subunit is highly expressed in numerous human malignant melanoma cells compared to normal epidermal melanocytes (Francia *et al*, 1999). Importantly, B56*γ* participates also in the regulation of the Wnt/*β*-catenin signalling, a pathway important in the control of cell proliferation and self-renewal of the cancer stem cell. In fact, B56*γ* directly interacts with APC in the *β*-catenin/axin/GSK-3*β* complex and induces *β*-catenin inactivation/degradation (Seeling *et al*, 1999). Moreover, the catalytic subunit of PP2A has been found associated to axin (Li *et al*, 2001), suggesting that PP2A:B56 is part of the *β*-catenin degradation complex.

In transformed cells, inactivation of PP2A phosphatase and, therefore, loss of its tumour suppressor activity may not only be a consequence of genetic alterations (i.e. mutations) and/or aberrant expression of a specific PP2A subunit, but it could also derive from aberrant activation of signal-transduction pathways leading to post-translational modifications of the PP2A catalytic subunits and/or increased expression of molecules that act as general physiological inhibitors of PP2A phosphatase activity (Janssens and Goris, 2001). In fact, activation of Jak2 (Yokoyama *et al*, 2001) and/or src (Chen *et al*, 1992) kinases results in the suppression of PP2A phosphatase activity through a mechanism that involves the phosphorylation of the PP2Ac subunits on tyrosine 307. Likewise, expression of pp32 (I1PP2A) and SET (I2PP2A), two potent physiologic PP2A inhibitors, suppresses the phosphatase activity of all holoenzyme forms of PP2A, most likely through direct binding to the PP2A catalytic subunit (Li *et al*, 1996; Janssens and Goris, 2001). Interestingly, SET (also known as I2PP2A, IGAAD or TAF-I*β*) is a nucleus/cytoplasm-localised phosphoprotein overexpressed in solid tumours and leukaemias (Fornerod *et al*, 1995; Carlson *et al*, 1998). In addition to its activity as an inhibitor of PP2A, SET is also a potent inhibitor of the tumour suppressor NM23-H1 (Fan *et al*, 2003). Notably, SET was described as part of a fusion gene with the nucleopore complex protein CAN in a patient with t(6;9)(p23;q34) acute myeloid leukaemia (AML) (Adachi *et al*, 1994), and it was found associated with the oncoprotein Mll (ALL1, HRX) in leukaemic cell lines (Adler *et al*, 1997). Thus, it is intuitive that genetic (mutations in the gene encoding a specific PP2A subunits) or functional (e.g. PP2Ac^Y307^ phosphorylation, SET overexpression) inactivation of PP2A may represent a step required for cancer development and/or progression. Indeed, the importance of the SET/PP2A interplay in cancer has been recently investigated in chronic myelogenous leukaemia in which a SET-mediated inhibition of PP2A phosphatase activity is distinctively occurring in patients with blast crisis CML (Neviani *et al*, 2005).

## BCR/ABL AND PP2A: THE YIN AND YANG OF BLAST CRISIS CML

Although the mechanisms responsible for CML disease progression are still largely unclear, growing evidence suggests that the phenotype of CML-BC cells depends on the unrestrained activity of BCR/ABL and on the genetic or functional inactivation of genes with tumour suppressor activity (Calabretta and Perrotti, 2004). Moreover, altered mRNA metabolism appears to play a pivotal role in blast crisis CML, as mRNA processing, export and translation of specific mRNAs controlling survival and differentiation of myeloid progenitors (e.g. Bcl-X_L_, GCSFR, C/EBP*α* and *β*, MDM2 and MYC) are aberrantly regulated by increased BCR/ABL expression through the activity of specific mRNA binding proteins (e.g. TLS/FUS, hnRNP A1, hnRNP E2, La, hnRNP K and CUGBP1) (Perrotti *et al*, 1998; Iervolino *et al*, 2002; Perrotti *et al*, 2002; Trotta *et al*, 2003; Guerzoni *et al*, 2006; Notari *et al*, 2006). Specifically, the mRNA export activity of hnRNP A1, an RNA-binding protein overexpressed in CML-BC, is required for cytokine-independent proliferation, survival and tumorigenesis of acute phase CML blasts and BCR/ABL-expressing myeloid progenitor cell lines (Iervolino *et al*, 2002). By Ribonomics (Tenenbaum *et al*, 2002), we identified SET as a novel BCR/ABL target whose mRNA is specifically associated with hnRNP A1 (Figure 1) in Ph^1^ K562 cells (Neviani *et al*, 2005).

### The SET/PP2A interplay in CML

SET expression is modestly affected in CML-CP^CD34+^ progenitors, but markedly upregulated in myeloid CML-BC^CD34+^ cells in which it correlates with loss of PP2A phosphatase activity (Neviani *et al*, 2005). Interestingly, PP2A inactivation is a direct consequence of increased expression of SET (Figure 1), which is induced by BCR/ABL in a dose- and kinase-dependent manner and, like BCR/ABL, progressively increases during transition to blast crisis (Neviani *et al*, 2005). In fact, imatinib treatment and SET downregulation restored PP2A activity back to normal levels. Interestingly, a portion of PP2Ac is phosphorylated on tyrosine 307 in BCR/ABL-expressing cells, and PP2A^TYR307^ levels decreases upon SET downregulation or inhibition of BCR/ABL activity (Neviani *et al*, 2005). Thus, it is possible that BCR/ABL also affects the ability of PP2A to reactivate itself by autodephosphorylation (Chen *et al*, 1992) and/or that the SET/PP2A association favours the recruitment of a BCR/ABL-activated tyrosine kinase, which inhibits PP2A through phosphorylation of the PP2Ac tyrosine 307. Alternatively, SET overexpression and src and/or Jak2 activity might independently contribute to PP2A inactivation in BCR/ABL cells (Figure 1). Moreover, the PP2A subunit PR65/A, which is also overexpressed in BCR/ABL^+^ cells, may also contribute to PP2A inactivation, as PR65/A overexpression could sequester the catalytic or variable subunits or act as a PP2Ac inhibitor (Kamibayashi *et al*, 1992; Wera *et al*, 1995). Interestingly, it was recently reported that hnRNP A2, an hnRNP with high sequence, structure and function similarity with hnRNP A1, inhibits PP2A activity upon interaction with SET (Vera *et al*, 2006). Because also hnRNP A2 expression is induced by BCR/ABL (our unpublished observation), the hnRNP A2/SET-dependent inhibition of PP2A may represent another mechanism whereby BCR/ABL induces the loss of PP2A function in CML-BC.

### Adverse molecular effects of BCR/ABL and PP2A

As reported above, the tumour-suppressing activity of PP2A depends on its ability to dephosphorylate several factors regulating cell cycle progression, proliferation, survival and differentiation (Sontag, 2001). Remarkably, several targets are shared by BCR/ABL kinase and PP2A phosphatase. Among these, expression and/or activity of the PP2A targets Myc, STAT5, MAPK, Akt, BAD and Rb (Neviani *et al*, 2005, and references therein) are either essential for BCR/ABL leukaemogenesis or have been found altered in CML-BC (Calabretta and Perrotti, 2004, and references therein). In BCR/ABL-expressing myeloid progenitor 32Dcl3 cells, inhibition of SET expression and, to a greater extent, forced expression of PP2Ac led to inhibition of MAPK, STAT5 and Akt phosphorylation, decreased Myc expression and increased levels of proapoptotic BAD (Neviani *et al*, 2005) (Figure 1). Since ectopic SET expression antagonised the effects of exogenous PP2A, it is possible that, in CML-BC progenitors, SET-dependent suppression of PP2A activity represents one of the main mechanisms used by BCR/ABL to prevent inactivation of mitogenic and survival signals required for its leukaemogenic activity. The importance of loss of PP2A activity for CML disease progression is also supported by the ability of PP2A to induce Rb dephosphorylation (Avni *et al*, 2003) and suppress Jak2 kinase, which was found activated in CML-BC (Xie *et al*, 2002).

### BCR/ABL as target of PP2A activity: role of SHP-1

In imatinib-sensitive and -resistant (T315I included) BCR/ABL cell lines and in CML-BC^CD34+^ patient cells, restoration of PP2A phosphates activity, achieved either by using chemical PP2A activators (e.g. forskolin; Feschenko *et al*, 2002) or by interfering with the SET/PP2A interplay (i.e. PP2Ac overexpression, SET knockdown), promotes BCR/ABL tyrosine dephosphorylation (inactivation) which, in turn, trigger its proteasome-dependent degradation (Neviani *et al*, 2005). The ability of PP2A to impair activity and expression of oncogenic tyrosine kinases is not unprecedented; in fact, PP2A induces inactivation and promotes proteolysis of src kinase (Yokoyama and Miller, 2001). Mechanistically, BCR/ABL proteolysis appears to depend on the PP2A-induced activation of the tumour suppressor SHP-1 tyrosine phosphatase and on the coexistence of BCR/ABL, PP2A and SHP-1 in the same multiprotein complex (Neviani *et al*, 2005) (Figure 1). In fact, increased cytokine-independent clonogenic potential and inability of PP2A to promote BCR/ABL degradation was observed in BCR/ABL-transduced SHP-1-decifient CD34^+^/lineage-negative marrow myeloid progenitors cells (Neviani *et al*, 2005). Interestingly, BCR/ABL dephosphorylation has been also observed in K562 cells overexpressing tyrosine phosphatase PTP1B (LaMontagne *et al*, 1998). Although ectopic PTP1B expression impairs clonogenicity and tumorigenesis of p210-BCR/ABL-transformed Rat-1 fibroblasts and induces K562 erythroid maturation (LaMontagne *et al*, 1998); it is still unclear why PTP1B expression is increased in BCR/ABL-expressing cells (LaMontagne *et al*, 1998) and whether PTP1B activity is altered in primary CML progenitors or changed during disease progression.

The involvement of SHP-1 in the PP2A-induced negative regulation of BCR/ABL kinase activity and expression is also supported by the fact that SHP-1 associates with BCR/ABL and its tyrosine phosphatase activity counteracts BCR/ABL leukaemogenic potential (Liedtke *et al*, 1998; Lim *et al*, 2000). Accordingly, expression of SHP-1 is diminished in most of leukaemias and lymphomas, SHP-1 downregulation leads to abnormal cell growth and SHP-1 activity is suppressed by different oncogenic tyrosine kinases (e.g. FLT3/ITD and JAK) (Wu *et al*, 2003; Chen *et al*, 2005a). Thus, functional inactivation of PP2A by increased BCR/ABL kinase activity seems to be required for the transduction of aberrant mitogenic, survival and antidifferentiation signals, and for the post-translational enhancement of BCR/ABL expression and oncogenic activity in CML-BC^CD34+^ marrow myeloid progenitors (Figure 1).

### Restoration of PP2A activity impairs BCR/ABL leukaemogenesis: therapeutic effects of the PP2A activator forskolin

Consistent with the negative effects of PP2A on BCR/ABL expression and function, restoration of PP2A activity back to normal levels via SET downregulation, PP2Ac overexpression or treatment with the potent PP2A activator forskolin (Feschenko *et al*, 2002) or with 1,9-dideoxy-forskolin (forskolin derivative that lacks adenylate cyclase-cAMP- (Seamon and Daly, 1981) activating function) induces marked apoptosis, reduces proliferation, impairs colony formation, inhibits tumorigenesis and restores differentiation of patient-derived myeloid CML-BC^CD34+^ cells and/or BCR/ABL-transformed cell lines, regardless of their degree of sensitivity to imatinib (Neviani *et al*, 2005). Notably, the imatinib-, AMN107- and dasatinib-resistant T315I BCR/ABL^+^ cells are also sensitive to PP2A activation (Neviani *et al*, 2005). Remarkably, forskolin but not 1,9-dideoxy-forskolin delayed proliferation of CD34+ normal marrow cells without inducing either apoptosis or PP2A activity.

Consistent with the *in vitro* effects of restoration of PP2A activity, *in vivo* administration of forskolin and/or 1,9-dideoxy forskolin severely impacted and efficiently modulated the development of wild-type and T315I BCR/ABL-induced acute leukaemia-like disease process in immunocompromised mice (Neviani *et al*, 2005).

Forskolin, a diterpene extracted from the roots of *Coleus forskohlii*, was primarily and extensively studied and used in rodents and humans for its adenylate cyclase-activating functions and, more recently, for its ability to activate PP2A. Although the molecular mechanism underlying PP2A activation by forskolin (Feschenko *et al*, 2002) in BCR/ABL-transformed cells is still largely unclear, it seems that it does not depend on increased cAMP or PKA activation (Neviani *et al*, 2005).

Clinical trials demonstrated the low toxicity of the water-soluble forskolin in the treatment of patients with cardiac dysfunction or bronchospasm (Wajima *et al*, 2003; Kikura *et al*, 2004). Furthermore, the use of forskolin as an antileukaemogenic agent is not unprecedented, as it inhibits growth and/or induces apoptosis of different types of leukaemic cells *in vitro* (Laneuville *et al*, 1992; Moon and Lerner, 2003). However, all of these studies were all focused on assessing the importance of cAMP in leukaemogenesis, as they were undertaken when the property of forskolin as PP2A activator was still unknown.

The *in vivo* forskolin and 1,9-dideoxy forskolin antileukaemic activity and the low toxicity are consistent with the long elimination half-life assessed in healthy individuals and with the absence of toxicity in mice treated with these compounds (Kikura *et al*, 2004; Neviani *et al*, 2005). Furthermore, the safety and therapeutic effects of these diterpenes are also suggested by the prolonged survival of SCID mice transplanted with wild-type or T315I BCR/ABL cells (50% of mice were alive after 25 weeks of treatment while untreated leukaemic mice died within 5 weeks), and by the lack of signs of toxicity or leukaemia after administration of these compound for 18 (Neviani *et al*, 2005) and 25 (our unpublished data) weeks at 4 and 8 mg kg^−1^ week^−1^.

Although the ability of forskolin to prevent tumour colonisation and metastasis was reported previously (Agarwal and Parks, 1983), our work for the first time underscores the therapeutic relevance of PP2A-activating drugs in cancer by specifically indicating that treatment of leukaemic mice with forskolin or 1,9-dideoxy forskolin remarkably prolongs the lifespan and decreases the leukaemia burden to a point in which no BCR/ABL^+^ cells are detected in peripheral blood of treated animals (Neviani *et al*, 2005).

## CONCLUSIONS

Because of the central role of PP2A in the regulation of cell growth, survival and differentiation, it is clear that its loss-of-function contributes to tumour development and progression. Moreover, the knowledge that functional inactivation of PP2A tumour suppressor activity occurs in myeloid blast crisis CML through the effect BCR/ABL on SET expression, and that re-establishment of normal PP2A activity antagonises both *in vitro* and *in vivo* BCR/ABL leukaemogenesis (see Figure 1), highlights the importance of incorporating PP2A activating drugs (e.g. forskolin) in the current therapeutic protocols for blast crisis and imatinib-resistant (T315I included) CML. In particular, the association of imatinib or new ABL-kinase inhibitors with drugs that enhances PP2A phosphatase activity may represent a successful therapeutic strategy for CML blast crisis patients that are nonresponsive or develop resistance to imatinib and, perhaps, for those patients with Ph1 ALL.
